# Flavor and appearance of whole shell eggs made safe with ozone pasteurization

**DOI:** 10.1002/fsn3.134

**Published:** 2014-06-13

**Authors:** Esther N Maxkwee, Jennifer J Perry, Ken Lee

**Affiliations:** 1 Department of Food Science and Technology, Ohio State UniversityOhio; 2 Stonyfield FarmLondonderry, New Hampshire; 3 Department of Food Science and Technology, Ohio State UniversityOhio

**Keywords:** Acceptance, egg, ozone, pasteurization, sensory

## Abstract

Raw eggs are a potential health hazard and a new federally approved process uses ozone to maintain freshness while ensuring safety. The impact of an ozone process on the flavor, color, and shell integrity of eggs must be known for market acceptance. The visual perception and consumer acceptance of full commercial scale ozone-pasteurized eggs are reported, using a degree of liking test and a Just-About-Right analysis. Instrumental analysis of albumen turbidity, yolk color, and Haugh Units correlate with human perception. Visual tests reveal that ozone-pasteurized eggs were equivalent to thermally pasteurized eggs in attributes such as appearance, spread, and cloudiness. They were equivalent to untreated eggs in yolk height, yellowness, and appeal. There were no differences in taste among all egg treatments for measures of aroma, flavor, texture and overall liking. Ozone-pasteurized eggs have the same appeal as raw eggs, and can be cooked without flavor loss. This promising new ozone process maintains good sensory quality relative to thermal processing.

## Introduction

Consuming food contaminated by pathogenic bacteria, viruses, or other organisms is a major problem worldwide. According to the Centers for Disease Control and Prevention, about 3000 Americans die each year because of foodborne diseases, with 130,000 hospitalized and 48 million sick (CDC 2011). *Salmonella* enteritidis is the illness caused by eating raw eggs. About 200,000 illnesses per year may be caused by *Salmonella* enteritidis contaminated eggs (Schroeder et al. 2005). In 2010, FDA (The US Food and Drug Administration) required egg producers to implement the final Egg Safety Rule, including refrigeration during storage and transportation (FDA 2010). The Food Safety Inspection Service (FSIS 2005) estimated that illnesses would decrease if all shell eggs were pasteurized to get a 5-log reduction. This lowering the number of organisms by 100,000-fold is commonly referred to as pasteurization (NACMCF 2005). To meet this national goal, researchers have examined methods to achieve a 5-log reduction of *Salmonella* enteritidis including irradiation, microwave, UV, thermal pasteurization, and heat–ozone pasteurization of shell eggs (Tellez et al. 1995; Davidson 2004; Lakins et al. 2008; Perry 2010).

Ozone has been used as a disinfectant since the 1800s (Weavers and Wickramanayake 2001). In 2001, FDA approved use of ozone in the treatment, storage, and processing of foods including meat and poultry, and as secondary food additive in gaseous or aqueous phases as an antimicrobial agent (FDA 2001). Ozone plus mild heat pasteurization, which is referred to simply as ozone pasteurization in this study, was shown to inactivate more than 5-log colony-forming units per g of *Salmonella* enteritidis in shell eggs (Perry and Yousef 2013). The ozone egg process gained regulatory approval in 2009 in anticipation of the design of the first large-scale commercial process.

Studies were conducted at Ohio State on the quality of small laboratory and pilot-scale ozone-pasteurized eggs before commercial scale equipment was conceived (Kamotani et al. 2010). Four factors drive this study – federal regulatory approval of ozone pasteurization, an investment by three major Ohio egg producers with proven consumer interest, the potential for ozone to cause oxidized flavor defects, and the award of a major commercialization grant to design and build large commercial scale ozone process machines. The commercial scale-up requires the sensory validation work here.

Pilot-scale ozone-pasteurized eggs appeared superior to thermally pasteurized eggs in a consumer acceptance study (Kamotani et al. 2010). There were no significant differences in degree of liking among three different egg treatments (thermal process, ozone pasteurized, and raw untreated) (Kamotani et al. 2010). Fourier transform infrared analysis of protein structure during extended storage suggested that ozone-pasteurized eggs were more likely to behave like untreated shell eggs. This is because albumen protein in ozone-pasteurized eggs is less damaged relative to thermally pasteurized eggs (Perry et al. 2011).

The design of industrial scale ozone process machinery achieves the same or better reduction in pathogens than pilot scale. As these eggs near market entry, it is critical to verify if the commercial process yields a same or better egg quality as perceived by consumers. This was achieved by the comparison of visual perception and consumer acceptance of ozone-pasteurized eggs versus thermally pasteurized eggs and raw untreated eggs. Standard industry measures of albumen turbidity, yolk color, and Haugh units were correlated with human measures. These data inform the industry and the consumer as potential market entry nears.

## Materials and Methods

### Eggs

All eggs in all three treatments throughout this study were US Grade AA large. Fresh untreated (raw eggs) and ozone-pasteurized eggs (ozone eggs) laid by hens between 32 and 45 weeks of age were shipped immediately after laying from the same Ohio farm and same flock by standard surface transport to a nearby egg distribution site. Thermally pasteurized eggs (heated eggs) were purchased, as it is commercially available, from a local grocery with a Julian date of 110 and evaluated 1 week later. All eggs were stored on standard fiber board egg trays inside a cardboard and held at a refrigerated temperature of 4°C until further use. Eggs from all sources were industrially washed, sorted, and any with cracks or checks discarded.

Unprocessed control eggs (raw eggs) received no further treatments; thus, they are identical to what is now most commonly purchased in groceries throughout the world. Heat–ozone-pasteurized eggs (ozone eggs) were processed at a commercial egg facility in Coldwater, Ohio following an FDA validated process, where gaseous ozone under pressure ensured a 5-log reduction of pathogens in the geometric center of each egg. Process conditions remain proprietary but were validated by a certified process control specialist. Thermally processed eggs (heated eggs) are treated with heat only up to 63°C for 40 min, as specified by Davidson (2004). The heated eggs were purchased from a local grocery under the brand name of Crystal Farms.

### Sensory panel

Sensory studies were done by the Ohio State University Food Science Sensory Group in a standardized test facility (Columbus, OH). Ten segregated booths were illuminated by 3000°K white fluorescent light and the temperature was kept constant at 21°C. All responses were collected using Compusense® version 5.2 software (Guelph ON, Canada) on a color LCD monitor interface controlled via mouse and keyboard by each panelist. Tests were conducted in one morning session in a complete balanced design, serial monadic approach for all three different egg treatments. Eggs were presented on black plastic plates, coded with three random digits, and mineral water provided between samples for mouth rinsing. Ninety-eight untrained panelists (73 females and 25 males, average age of 34 years) were recruited from a roster of consumers maintained by the OSU Sensory Analysis Laboratory. Over 90% of the panelists reported eating eggs regularly, 25% ate two per week, 12% ate three per week, and 13% ate four per week. Prior to testing, it was verified that participants had no egg allergies or aversions. Ethnicity was 4% Asian, 7% Black, 3% Hispanic, 81% White, 2% other, and 3% preferred not to disclose.

### Consumer rating of egg appearance

**Figure 1:**
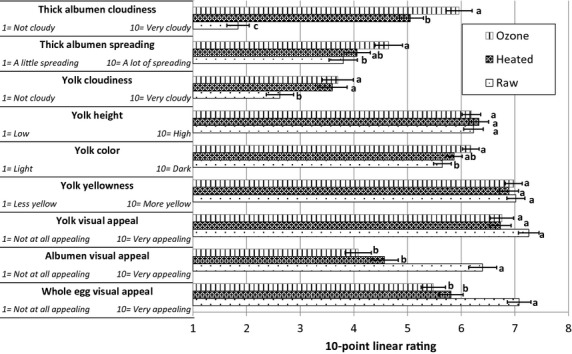
Visual ratings of three egg treatments by 98 consumers viewing the freshly cracked, uncooked contents over a black matte background. Ratings are a linear 10-point scale with the endpoint descriptive anchors at 1 and 10 listed at left. (a–c) Within each group of three bars a different letter indicates a significant difference at *P* > 0.05. Error lines show one standard error of the mean.

Randomly selected eggs from each of three treatments (ozone, heated, and raw) were cracked into a clear 15.9-cm plate (The Kroger Co., Cincinnati, OH), labeled with three-digit random numbers, and placed over a black matte paper background to test visual acceptance. Each panelist was asked to individually rate samples on a 10-point linear scale for these nine different attributes: 1 = *cloudiness of the thick albumen*, 2 = *amount of spreading of thick albumen*, 3 = *cloudiness of the egg yolk*, 4 = *height of the yolk*, 5 = *color of the yolk*, 6 = *yellowness of the yolk*, 7 = *visual appeal of the yolk*, 8 = *visual appeal of the albumen*, and 9 = *visual appeal of the whole egg*. Panelists viewed the freshly cracked uncooked eggs in random order within 1 h of removing the shell. The endpoint anchors at rating 1 and 10 for each attribute were labeled as shown in Figure 1.

### Consumer rating of cooked eggs

In a consumer acceptance test, six eggs for each treatment were premixed a day before the test using a hand blender with a whisk attachment (Model #59780, Hamilton Beach, Washington, NC) on high for 45 sec. Each premixed egg sample was placed into labeled Ziploc bags and stored in a commercial refrigerator (Delfield Refrigerator, Mt. Pleasant, MI) at 4°C until further use. On test day, each bag of egg mix was placed into a microwave safe glass bowl and mixed on high for 10 sec. The egg mixes were cooked in a microwave (Model JES1358WL 01, General Electric, Louisville, KY) for 1.0 min on high at 1100 W. The eggs were then mixed on low for 10 sec and cooked for an additional 1.0 min. The eggs were cut into large pieces (∼5 cm^2^) using a #1905 rubber spatula (Rubbermaid, Fairlawn, OH) and cooked for an additional 1.0 min. Finally, the eggs were cut into smaller pieces (∼2 cm^2^) using the spatula and covered with cling wrap (Wasserstrom, Columbus, OH) for 30 sec. Temperature was measured with a handheld thermometer (Rubbermaid) until a consistent internal temperature of 73.9°C was reached for all treatments. Cooking across all treatments was consistent. Each batch was placed in equal portion (about 45 g) onto ten 15.2 cm disposable white foam plates (Supreme Pactiv Corp., Lake Forest, IL) using a 46.1 g scoop (NSF, Sheboygan, WI). Filtered spring water was provided to the panelists as their palate cleanser between samples.

Panelists were instructed to individually rate the samples for degree of liking on a 9-point hedonic scale, where 9 = like extremely, 8 = like very much, 7 = like moderately, 6 = like slightly, 5 = neither like nor dislike, 4 = dislike slightly, 3 = dislike moderately, 2 = dislike very much, 1 = dislike extremely.

**Figure 2:**
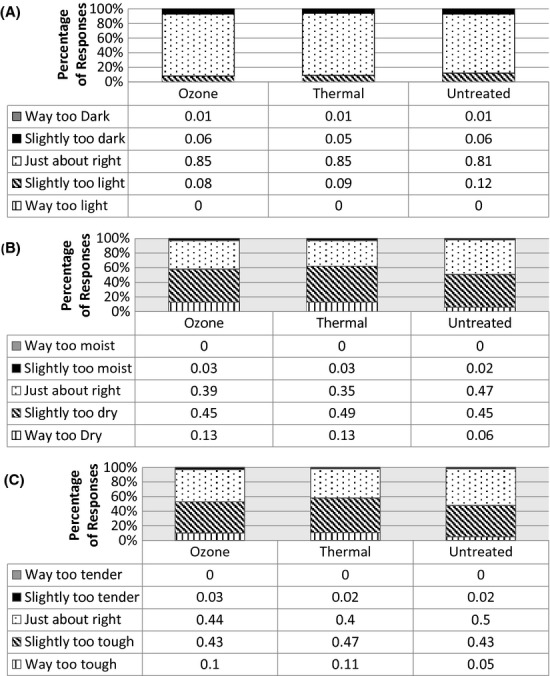
Consumer scores (*n* = 98) on a Just-About-Right (JAR) rating of scrambled eggs by three treatments for (A) Color, (B) Moistness, and (C) Texture.

The hedonic attributes tested were visual, aroma, flavor, texture, and overall liking. These same panelists were instructed to use a 5-point category Just-About-Right (JAR) scale to rate the amount of scrambled egg color, moistness, and texture. The descriptors for the JAR analysis across all three egg treatments are listed in Figure 2. Finally, demographic data were obtained on each panelist, including age, gender, egg usage, and race.

### Instrumental analyses

Instrumental analyses measured differences between ozone eggs versus raw eggs, both from the same flock at the same time. Albumen turbidity measurements were based on 12 replicates. Haugh units and yolk color measurements were based on 10 replicates. Albumen turbidity measurement was performed by separating the albumen from the yolk using an egg separator (MSC International, Montreal, Canada). Three milliliter of the albumen were pipetted into disposable cuvettes. Absorbance was measured at 600 nm with a Shimadzu UV-Vis 2450 spectrophotometer (Kyoto, Japan) and samples were read against a blank reference of distilled water (Perry et al. 2011). Twelve measurements were taken for each treatment and a standard deviation was calculated.

Haugh units (HU) were measured using a HU meter (Mattox & Moore, Indianapolis, IN, USA) and is based on the following formula HU = 100 log_10_ (h − 1.7w^0.37^+ 7.6), where HU is the Haugh units, h is the thick albumen height, and w is the weight of the unbroken egg (Perry et al. 2011). Egg weight of 680 g per dozen was set manually. Eggs were cracked onto a leveled surface; the meter was placed over the egg with the middle pin positioned approximately in the middle of the thick albumen, avoiding the chalazae. Measurements from the dial were recorded when the middle pin touched the albumen. Averages from two different measurements were taken in two different areas of the albumen. Ten eggs were measured for each treatment and a standard deviation was obtained.

The yolk color was measured using a Colorquest XE Hunter Lab colorimeter (Reston, VA) with Illuminant D65/2 and 2.54-cm-diameter viewing aperture (adapted from Pihlsgard and Renee 2007). Yolk was separated from the albumen. Ten milliliter of yolk was placed into a cuvette. Ten measurements of L*, a*, and b* values were recorded.

### Statistical analyses

Significant differences within consumer acceptance tests were determined using repeated measures analysis of variance and Tukey's HSD post hoc analysis at *P* < 0.05, generated from Compusense software. Significant difference analysis on instrumental analyses was performed using independent t-test generated from SPSS Statistics version 16.0 software (SPSS Inc., Chicago, IL).

## Results and Discussion

### Consumer rating of egg appearance

Cloudiness of the albumen is a visual indication of egg freshness. Fresh eggs are cloudier than older eggs because carbon dioxide inside eggs escapes over time, thus creating albumen that is more translucent (AEB 2010). As shown in Figure 1, a consumer panel rated the thick albumen of ozone-pasteurized eggs (hereafter, ozone eggs) as significantly cloudier than thermally pasteurized eggs (heated eggs). They also rated the heated eggs significantly cloudier than untreated raw eggs (*P* < 0.05). This result is similar to an earlier pilot-scale study conducted by Kamotani et al. (2010). These results are consistent with instrumental measures. As shown in Table 1, instrumentally measured albumen turbidity of ozone eggs was significantly higher than the raw egg (*P* < 0.05), confirming the consumer panel observations.

**Table 1:** Physical properties of egg quality before (raw) and after processing (ozone). Haugh Unit expressed as HU = 100 log_10_ (h − 1.7w^0.37^ + 7.6) is increased, while tristimulus colorimeter measures of value, hue and chroma, or L*, a*, b*, respectively, show no significant color change. Turbidity by optical density increased.

Physical property	*N*	Before processing, raw	After ozone processing
Haugh unit, HU	10	85.6 ± 2.700^b^	92.2 ± 1.220^a^
L* value	10	61.7 ± 0.440^a^	62.4 ± 0.370^a^
a* hue	10	12.2 ± 0.230^a^	12.2 ± 0.290^a^
b* chroma	10	57.1 ± 0.600^a^	57.5 ± 0.340^a^
Turbidity, OD	12	0.020 ± 0.010^b^	0.321 ± 0.040^a^

Mean ± standard error of mean. Within each row, different letters indicate a significant difference at α = 0.05.

Perry et al. (2011) reported different results in a pilot-scale study. They found that albumen turbidity from ozone eggs was significantly higher than that of raw eggs as we did here, but the ozone eggs were significantly less turbid than heated eggs. This is likely due to a different thermal pasteurization technique from the Perry study and a difference in purchased thermally pasteurized eggs.

A common egg-grading and consumer acceptance criterion is the spreading of the thick albumen. Eggs with the least spreading of the thick albumen earn higher grades than those with more spread (AEB 2010). The spreading of the heated egg thick albumen was not significantly different when compared to ozone eggs and raw eggs. Ozone and raw eggs were significantly different from each other, where ozone eggs were perceived to have more spreading (mean rating of 4.65) than raw eggs (mean rating of 3.80 in Fig. 1). Our previous study with pilot-scale egg treatments showed no significant differences between the ozone and raw eggs, but heated eggs were perceived to have significantly less spread (Kamotani et al. 2010).

The standard for grading of egg quality is the Haugh Unit, devised by Raymond Haugh in 1937 and in continuous industry and regulatory use. HU measures the height of the thick albumen. Because spreading of the albumen directly correlates with the height, a higher HU indicates thicker albumen and less spread. Eggs that have greater than 72.0 HU are grade AA (USDA 2000). Table 1 shows that the average HU of ozone eggs was 92.2, which was significantly higher than that of the raw eggs (85.6). This shows that the ozone egg treatment yields a higher quality HU measurement than untreated. Nonetheless both the raw and ozone eggs have a HU >72 that qualifies them as grade AA.

Yolk color is not a grading criterion but affects consumer perception. Ozone eggs were perceived by consumers to be darker in color than raw eggs shown by a 6.18 mean rating score compared to 5.65 mean rating score. All three egg treatments were perceived to have no significance difference in their yellowness, as shown in Figure 2. In our pilot treatment study, there were no differences across all egg treatments in lightness but the heated eggs were perceived to be less yellow. There were no significant differences between ozone eggs and raw eggs (Kamotani et al. 2010). This is consistent with the physical property measures shown in Table 1, where ozone eggs and raw eggs have no significant differences in their hue, value, or chroma.

The effect of ozone processing on the strength of the eggshell was measured by Instron analyses across seven different batches of eggs laid over a period of 6 months and measured as a function of storage at 0, 6, and 12 days. There was no significant difference among batches of ozone-pasteurized eggs regardless of storage days or treatment batches. As expected, significantly more force was required to crack untreated versus ozone eggs (*P* < 0.05) as the treatment slightly weakens the eggshell. These data are not shown as the eggshell has no effect on the sensory acceptance of the egg contents when presented visually or as cooked scrambled eggs.

With respect to the cloudiness of the egg yolk, the ozone eggs and heated eggs were not significantly different from each other, but both were perceived as significantly cloudier than raw eggs. Ozone eggs were rated significantly different from heated and raw eggs (Kamotani et al. 2010). This suggests that commercially processed ozone eggs are better than heated eggs in yolk cloudiness, and are also better than the pilot scale eggs in their appearance. As shown in Figure 1, all eggs were rated below 4 on the intensity scale of 1 being not cloudy and 10 being very cloudy. Thus, yolk cloudiness should not be a major consumer acceptance concern.

The height of egg yolk is an important criterion for egg grading. Yolk that is round and upstanding is graded AA or A, while yolk that is enlarged and flattened is graded B (AEB 2010). In the visual test shown in Figure 1, there was no significant difference (*P* > 0.05) in the height of the yolk among ozone, heated, and raw eggs. Conversely, ozone eggs were perceived to have a shorter yolk when compared to other treatments (Kamotani et al. 2010). This shows improved yolk quality since ozone eggs are perceived equivalent to heated and raw eggs. The yolk index of the ozone eggs was not significantly different than raw eggs between 2 and 8 weeks of storage (Perry et al. 2011). All eggs after processing by heat or ozone continued to meet grade AA standards.

Regardless of varying perception of yolk attributes, the visual appeal of the yolk was not different among all three egg treatments with a rating of about 7 in intensity scaling, where 10 was very appealing (Figure 1). Queries about the visual appeal of the albumen and whole egg showed that the ozone eggs and heated eggs were similar, but both were perceived less appealing than raw eggs. Thus, the visual appeal of ozone egg is indistinguishable from heated eggs.

### Consumer rating of cooked eggs

While visual tests show varied perceptions of ozone, heated, and raw eggs, taste tests of scrambled eggs from these three treatments did not vary. Figure 1 shows that all three egg treatments, when prepared from a consistent scrambled egg recipe, were liked slightly with a hedonic rating of 6 for all five attributes (visual, aroma, texture, flavor, and overall liking). There were no significant differences across all treatments in all attributes. The scrambled eggs were liked slightly, a rank commensurate to being served plain, unseasoned, and unaccompanied. Seasoning the scrambled eggs may have increased liking, but would also mask, not accentuate any differences among treatments.

A proprietary focus group study was conducted by CMA Consulting (Heavenridge 2013). Five professional chefs prepared several egg dishes and their observations were recorded. Scrambled eggs prepared with ozone eggs were judged equivalent or better to raw eggs. In the focus group, the chefs observed that the ozone egg yolk tasted very good and had an appealing bright yellow color.

Several Just-About-Right (JAR) results are presented in Figure 2. More than 80% of panelists perceived all three-treatment scrambled egg color to be the median rank of 3, just about right. This is an improvement from earlier work that reported ozone eggs were perceived as slightly too light relative to other treatments (Kamotani et al. 2010).

When panelists were asked to rate the amount of moistness on a JAR scale, heated eggs were perceived as slightly drier than the ozone or raw treatments. In the attribute of moistness, both heated and ozone eggs received more “way too dry” rankings than raw eggs. When panelists were asked to rate the egg texture on the JAR scale, heated eggs were slightly tougher than the other two treatments, while raw eggs received the most “just about right” ratings in the texture attribute among all eggs. Heated eggs had the highest number of responses in the “slightly” and “much too dry” category as shown in Figure 2.

In correlation with the liking results, the dryness and toughness were attributable to the microwave method of cooking. A conventional stovetop yields less dry and less tough scrambled eggs, but the large variation in the conventional cooking method would mask differences between treatments. These results are consistent with prior work where there were no differences in JAR ratings for moistness and texture among treatments. Both methods of pasteurization of eggs were perceived to be slightly too dry and too tough (Kamotani et al. 2010).

## Conclusion

Commercially pasteurized and regulatory approved ozone eggs have the same or better visual appeal as heated pasteurized eggs. In some cases, including height, yellowness, and visual appeal of the yolk, ozone eggs were perceived equivalent to raw eggs. In taste tests, there were no significant differences in score among all egg treatments for the visual, aroma, flavor, texture, and overall liking when presented and tasted as scrambled eggs. Sensory test results indicate that yolk quality has improved in the commercial process relative to a pilot-scale process, where the yolk quality of the commercially processed ozone eggs is perceived equivalent to that of heated eggs. Ozone eggs had an average of 92.2 HU, well above that needed to qualify this process as grade AA. Commercially processed ozone eggs achieve the safety required by the US Egg Safety Action Plan. These sensory tests show that they achieve equivalent consumer acceptance to the traditional raw shell egg and are appropriate for in-home or institutional cooked egg recipes.
